# Mucosal Hallmarks in the Alimentary Canal of Northern Pike *Esox lucius* (Linnaeus)

**DOI:** 10.3390/ani10091479

**Published:** 2020-08-22

**Authors:** Giampaolo Bosi, Massimo Lorenzoni, Antonella Carosi, Bahram Sayyaf Dezfuli

**Affiliations:** 1 Department of Health, Animal Science and Food Safety, Università degli Studi di Milano, St. Trentacoste 2, 20134 Milan, Italy; giampaolo.bosi@unimi.it; 2 Department of Cellular and Environmental Biology, University of Perugia, St. Elce di sotto 5, 06123 Perugia, Italy; massimo.lorenzoni@unipg.it (M.L.); antonella.carosi@unipg.it (A.C.); 3 Department of Life Sciences and Biotechnology, University of Ferrara, St. Borsari 46, 44121 Ferrara, Italy

**Keywords:** carnivorous fish, digestive tract, endocrine cells, immunofluorescence, mucous cells

## Abstract

**Simple Summary:**

In vertebrates, mucous cells are one of the main cellular components of the gut mucosal system, which secrete different mucin types involved in several functions. Endocrine cells are scattered in the epithelium of the gut mucosa, and they produce and release regulatory molecules affecting food intake and nutrition. The goal of this study was to obtain data on quantitative distribution of mucous and endocrine cell types in the alimentary canal of the northern pike (*Esox lucius*), using histochemistry and immunofluorescence. In the stomach of pike, there is a high abundance of mixed mucins, with the acid component contributing to the lubrication of mucosae, where they are associated with the rapid passage of digesta through the intestine. Neutral mucins increase in the intestine aborally. The distribution of endocrine cells of the diffuse endocrine system shows the presence of somatostatin and catecholamine-secreting endocrine cells and the lack of gastrin-secreting endocrine cells. We show a close regulatory relation between endocrine and mucous cells of the gut mucosal system involved in the physiology of fish nutrition. Results confirmed the relationship between the carnivorous diet and the gut mucins distribution of northern pike; indeed, our data provide very important information to ichthyologists who study dietary behavior of species.

**Abstract:**

On the basis of trophic behavior, fish are classified as herbivores, carnivores, omnivores, or detritivores. Epithelial mucous cells secrete mucin types specific to diet and digestive function. Mucus secretion is regulated mainly by molecular modulators produced by epithelial endocrine cells in response to luminal or tissue stimuli. These modulators are involved in control of food intake and digestive functions. Immunohistochemical and immunofluorescence studies were conducted on 10 adult northern pike (*Esox lucius* Linnaeus, 1758) from Lake Piediluco (Central Italy) to quantify distribution of sub-types of mucous and endocrine cells in alimentary mucosal epithelium. Neutral mucins predominated in the esophagus, and mixed and acidic mucins predominated in stomach and intestine. The gastric epithelium contained endocrine cells secreting somatostatin, tyrosine hydroxylase, and substance P. Mucous cells secreting neutral mucins increased in number from proximal to distal intestine, with endocrine cells containing substance P in the proximal intestine and those containing Leu-enkephalin throughout the intestine. Lectin histochemistry of gut sections revealed an abundance of N-acetyl-glucosamine and N-acetyl-galactosamine as carbohydrate residues on the mucin chain. The quantity and content of endocrine and mucous cells in the alimentary canal of *E. lucius* showed a direct relationship with its diet.

## 1. Introduction

Studies of morphology and function of the fish digestive system are key to understanding aspects of their ecology and adaptations to habitat [1]. The northern pike, *Esox lucius* Linnaeus, 1758, is a common ambush predator of freshwater habitats in the Palearctic [2,3]. Gross morphology of its alimentary canal was published by Bucke [2], who reported distribution and quantity of mucous cells altered by severe cestode *Triaenophorus nodulosus* (Pallas, 1781) infection. Later accounts described qualitative distribution of mucous cells in the gut [4].

Mucous cells are an essential component of the gut mucosal barrier, continuously producing and secreting mucins at the epithelial surface [5,6]. Most studies of the distribution of mucous cell types in fish gut are based on histochemical reaction to alcian blue (pH 2.5) followed by periodic acid Schiff (AB/PAS) staining or on high iron diamines staining followed by alcian blue (pH 2.5) (HID/AB) [6,7,8]. Lectin histochemistry allows better characterization of carbohydrate residues in the chemical structure of secreted mucins [9,10,11,12]. Lectins show binding affinity specific to sugar residues and can be used as markers to localize carbohydrate residues in glycoconjugates [13]. Recently, lectins have been used as biomarkers to detect specific secretory functions, structural components, and alterations in cells and tissues [7,11,12,14].

In fish, gut physiology is controlled by the spinal autonomic and enteric nervous systems [15]. Additionally, the cells of the diffuse endocrine system, randomly scattered among epithelial cells, regulate the function of the local gastrointestinal mucosa [16]. The gut endocrine cells produce about 30 different neuroendocrine substances, some of which are involved in appetite/satiety control, providing orexigenic/anorexigenic signals [17].

Our goal was to characterize the mucosal system of the alimentary canal of *Esox lucius* by mapping the distribution of mucous and endocrine cell types using traditional histochemical reactions, lectin histochemistry, and immunofluorescence.

## 2. Materials and Methods

The alimentary canals of 10 *E. lucius* (66.7 ± 9.7 cm, mean total length ± SD) from Lake Piediluco (Terni Province, Central Italy) were examined. The fish were collected by trapping by the Piediluco fishing consortium in May 2019 and transferred live to the consortium’s facilities, where they were euthanized with an overdose of 125 mg/L tricaine methanesulfonate (Sandoz, Basel, Switzerland). Fish were dissected and the alimentary canal was immediately removed and immersed in phosphate-buffered saline (pH 7.4; Sigma-Aldrich, Milan, Italy). Seven parts were distinguished: esophagus; esophagus–stomach junction; proximal and distal stomach; and proximal, medial, and distal intestine. Fragments (15 × 15 mm) of each were fixed in 10% neutral buffered formaldehyde solution. After 24 h, fixed samples were processed by routine paraffin embedding and cut into 4–6 µm sections.

Sections were stained with alcian blue 8GX (pH 2.5)/periodic acid Schiff (AB/PAS) and high iron diamines/alcian blue 8GX (pH 2.5) (HID/AB) sequences and subjected to lectin histochemistry. The AB/PAS stain method distinguishes among acidic (blue), neutral (magenta), and mixed neutral/acidic (purple-violet) mucins. The HID/AB sequence allows discrimination between acidic sulfated (brownish-black) and acidic carboxylated non-sulfated mucins (blue). Lectin histochemistry followed the procedure previous described [12,14]. The lectins used, source codes, and primary sugar affinities are reported in Table 1. Sections were treated with each lectin and 0.2 M solution-associated sugars (Table 1) in order to inhibit lectin reactivity.

For immunohistochemistry, dewaxed and re-hydrated sections were processed by standard procedures [14]. A panel of antibodies to components of the enteric neural and diffuse endocrine systems were used. These antibodies were previously tested on the digestive tract of several fish species [18,19,20] and, in *E. lucius*, on the neural structure of the myenteric plexus of the gut (data not shown). This study focused on the mucosal features of the *E. lucius* gut, and therefore we considered only the four markers reactive to endocrine epithelial cells of the diffuse endocrine system of the digestive tract of *E. lucius* (Table 2). Antibodies, sources, and dilution and incubation parameters are reported in Table 2.

The immunohistochemical reactions were validated with a different antibody for each identified molecule (Table 2). Mammalian tissue sections were used as positive controls. Some sections were analyzed by both immunohisto- and histo-chemistry to demonstrate mucin differential distribution. Following immunohistochemical analysis, we treated the sections with AB/PAS [19].

Histochemical- and immunohistochemical-treated sections were examined and photographed under an Olympus BX51 microscope (Olympus, Milan, Italy) equipped with a digital camera (Camedia C-5160, Olympus, 5.1 Mp) and image analysis software (DP-soft, version 3.2, Olympus). For quantitative histochemical studies, we photographed five fields from three sections of each of the main gut regions (150 samples per region) per fish at 40× objective (Olympus, N.A. 0.75) magnification. Mucous cells containing acid, neutral, or a combination of acidic and neutral mucins were distinguished and counted. In the sections stained with HID/AB, we counted the mucous cells containing sulfated and carboxylated mucins in the same manner. This procedure was also used to count mucous cells positive for each lectin and the antibody-positive endocrine cells in the gut. Mucous and endocrine cell counts were reported as mean number ± standard error (SE) per 100,000 μm^2^ of epithelial area.

Three double-immunofluorescence reactions were conducted on stomach sections to reveal possible co-expression of anti-somatostatin-14, anti-tyrosine hydroxylase, and anti-substance P. Dewaxed and rehydrated sections were treated with 1:20 normal goat serum in 0.05 M Tris-HCl (pH 7.4) and 0.55 M NaCl (TBS) for 30 min at room temperature, and subsequently with the Avidin-Biotin Blocking Kit (Vector Labs, Burlingame, CA, USA) according to the manufacturer’s instructions. After washing 2 × 5 min in TBS, sections were incubated with the first primary antibody (Table 2) for 72 h at 4 °C, with the secondary biotinylated antibody (Table 2) for 2 h at room temperature, and finally with 10 µg/mL fluorescein avidin D (Vector Labs) in 0.1 M NaHCO_3_ (pH 8.5) with 0.15 M NaCl for 2 h at room temperature. Slides were washed twice in TBS for 5 min and incubated with the second primary antibody (Table 2) for 72 h at 4 °C. Sections were subsequently treated sequentially with the secondary biotinylated antibody (Table 2) for 2 h at room temperature and with 10 µg/mL rhodamine avidin D (Vector Labs) in 0.1 M NaHCO3 (pH 8.5) with 0.15 M NaCl for 2 h at room temperature. The stained sections were mounted with Vectashield mounting medium (Vector Labs) and examined with the Olympus FW300 confocal laser scanning microscope (Olympus), equipped with multi-argon/helio neon green lasers and filters set for fluorescein and rhodamine. Confocal image acquisition and analysis were performed with Fluoview software version 5.0 (Olympus). For double immunofluorescence, we used pairs of antibodies from different hosts (Table 2). Cross-contamination of the signals was prevented by alternate excitation (0.2 s^−1^) at 488 nm (multiargon laser) and 540 nm (He–Ne green laser).

## 3. Results

In the alimentary canal of the pike, the histometric measurements indicated a major height of mucosal folds in the intestinal regions in comparison to the other tracts of the gut (Table 3). The height of the epithelium showed a major thickness in the esophagus and proximal intestinal region (Table 3).

**Figure 1:**
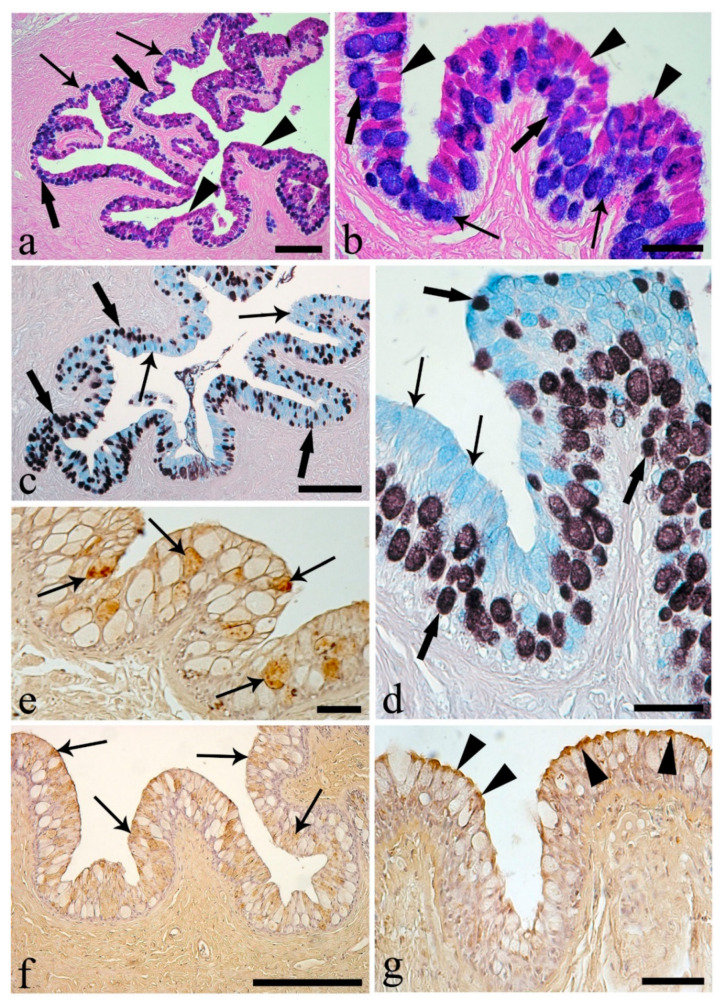
Esophagus mucosa. (**a**) Epithelium with numerous mucous cells containing neutral (arrowheads, PAS-positive), acid (thin arrows, AB-positive), and mixed (thick arrows, AB/PAS-positive) mucins. Scale bar: 200 μm. (**b**) Mucous cells almost equal to thickness of the epithelium, with neutral mucins (arrowheads) primarily in outer layer and with acid (thin arrows) and mixed (thick arrows) mucins in the basal part of the epithelium. Scale bar: 50 μm. (**c**) Mucous cells containing mainly carboxylated (thin arrows, AB-positive) and with fewer sulfated (thick arrows, HID-positive) mucins. Scale bar: 200 μm. (**d**) Mucous cells with carboxylated mucins (thin arrows) predominant in the upper part of the epithelium; mucous cells with sulfated mucins (thick arrows) in the basal area. Scale bar: 50 μm. (**e**) Mucous cells positive to the lectin dolichos biflorus agglutinin (DBA; thin arrows). Scale bar: 50 μm. (**f**) Mucous epithelial cells (thin arrows) positive to lectin wheat germ agglutinin (WGA). Scale bar: 200 μm. (**g**) Brush border positive to the lectin concanavalin A (ConA; arrowheads). Scale bar: 50 μm.

With the exception of the stomach, the esophageal epithelium showed the highest mean number of mucous cells (Table 4), with a predominance of PAS-positive and equal quantities of AB- and AB/PAS-positive mucous cells (Table 4). The esophageal acidic mucous cells contained 57.6% carboxylic and 42.4% sulfated mucins (Table 5). The deeper mucous cells of the esophageal epithelium were predominantly AB- or AB/PAS-positive, whereas, at the apical surface, the majority were PAS-positive (Figure 1a,b). The HID-reactive mucous cells, containing acid sulfated mucins, were mainly observed in the basal area of the epithelium, with the weak AB-reactive mucous cells containing carboxylated mucins at the apical area (Figure 1c,d).

A proportion of mucous cells of the esophagus were weakly positive to two of the six lectins, dolichos biflorus agglutinin (DBA) and wheat germ agglutinin (WGA) (Figure 1e,f). A mean of 61.1 ± 2.5 per 100,000 µm^2^ mucous cells in the esophagus were positive to DBA (17.9%). The mean number of WGA-reactive mucous cells was 210.3 ± 5.6 per 100,000 µm^2^, 50.5% of the mucous cells in the esophagus. The epithelial brush border showed reactivity to the lectin concanavalin A (ConA) (Figure 1g).

**Figure 2:**
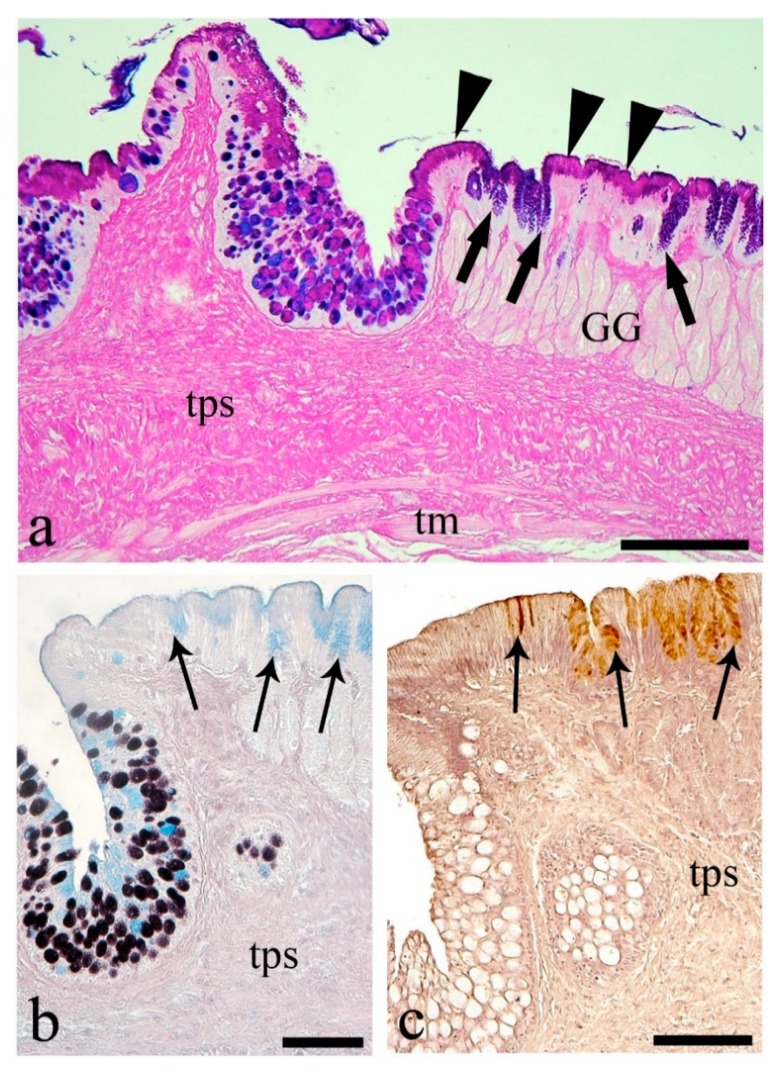
Esophagus to stomach. (**a**) Transitional area showing the thickness of the gastro-esophageal wall. Mucous cell distribution in the esophagus already seen in Figure 2. Mainly PAS-positive (arrowheads) surface mucous cells of the stomach; in the pits, cells contain mixed mucins (thick arrows). Note the unstained gastric glands (GG), the *tunica propria-submucosa* (tps), and the *tunica muscularis* (tm) with mixed striated and smooth muscle fibers. Scale bar: 200 μm. (**b**) HID/AB shows the same cell distribution as in Figure 1 for the esophagus, the lack of mucous cells with sulfated mucins, and the occurrence of mucous cells weakly positive to AB (thin arrows) in stomach pits. Scale bar: 100 μm. (**c**) Apex of stomach epithelium showing reactivity of mucous cells to lectin peanut agglutinin (PNA) (thin arrows). Scale bar: 100 μm.

At the junction of the esophagus and stomach, the mucous cells of the surface epithelium were primarily PAS-positive and were AB/PAS-reactive in the epithelium of gastric pits, while gastric glands were unstained (Figure 2a). The epithelium of this region was nonreactive to HID, and stained weakly with AB in the gastric pits, while gastric glands were unstained with HID/AB (Figure 2b). Mucous cells containing sulfated acidic mucins (HID-positive) were present in the esophageal mucosa but not in the stomach mucosa (Figure 2b). Only the peanut agglutinin (PNA) lectin gave a positive signal at the apex of the epithelial mucous cells of the junction of the esophagus and stomach (Figure 2c).

**Figure 3:**
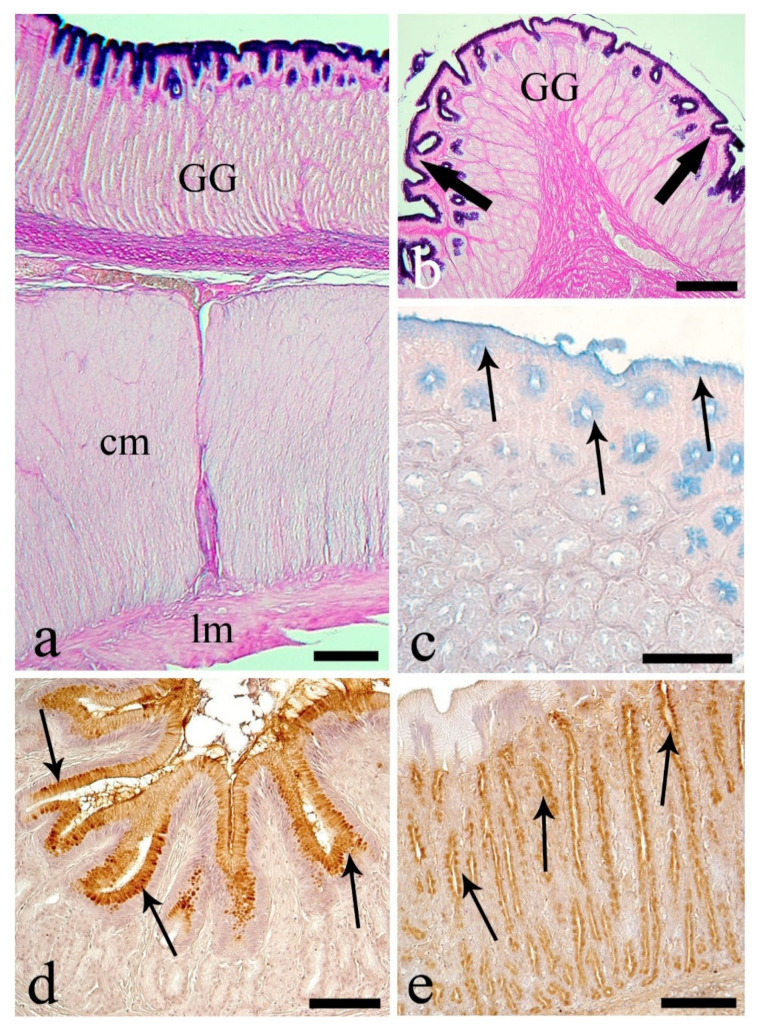
The stomach. (**a**) Longitudinal section shows two thick layers, the gastric glands (GG) and the *tunica muscularis* with circular (cm) and longitudinal (lm) muscle. Scale bar: 200 μm. (**b**) Transverse section of a longitudinal fold in the proximal region where apex shows mucous cells, mainly AB/PAS-positive (thick arrows); gastric glands are unstained. Scale bar: 200 μm. (**c**) Absence of mucous cells with sulfated mucins, and carboxylated mucins stained with AB (thin arrows). Scale bar: 100 μm. (**d**) Mucous cells at the luminal surface of the proximal region positive to the lectin PNA (thin arrows). Scale bar: 100 μm. (**e**) Apical portion of gastric glands in the distal region showing reactivity to lectin WGA (thin arrows). Scale bar: 100 μm.

In cross-section, the stomach wall appeared thicker compared to the esophagus (Figure 3a), with a wide glandular region and two distinct muscle layers of differing thickness with internal fibers circular and the external longitudinal (Figure 3a). The majority of the stomach mucosa took the form of longitudinal folds appearing sharply curved in transverse section (Figure 3b) and containing typical alveolar gastric glands (Figure 3b,c). The quantity of mucous cells in the stomach was higher than in any other part of the digestive tract (Table 4) and contained 68.7% of mixed- and 31.3% of PAS-positive mucous cells (Table 4), whereas the gastric glands were unstained (Figure 3a,b). The HID/AB stain showed only weakly AB-positive mucous cells with carboxylated acidic mucins at the epithelium apex (Figure 3c, Table 5). In the proximal stomach, the epithelial cells were positive to PNA (Figure 3d), similar to the area of transition from the esophagus to the stomach. In the distal stomach, the mucosa was flattened and thinner in transverse section. The gastric glands were tubular (Figure 3e) and showed a positive reaction to lectin WGA at their apices (Figure 3e).

**Figure 4:**
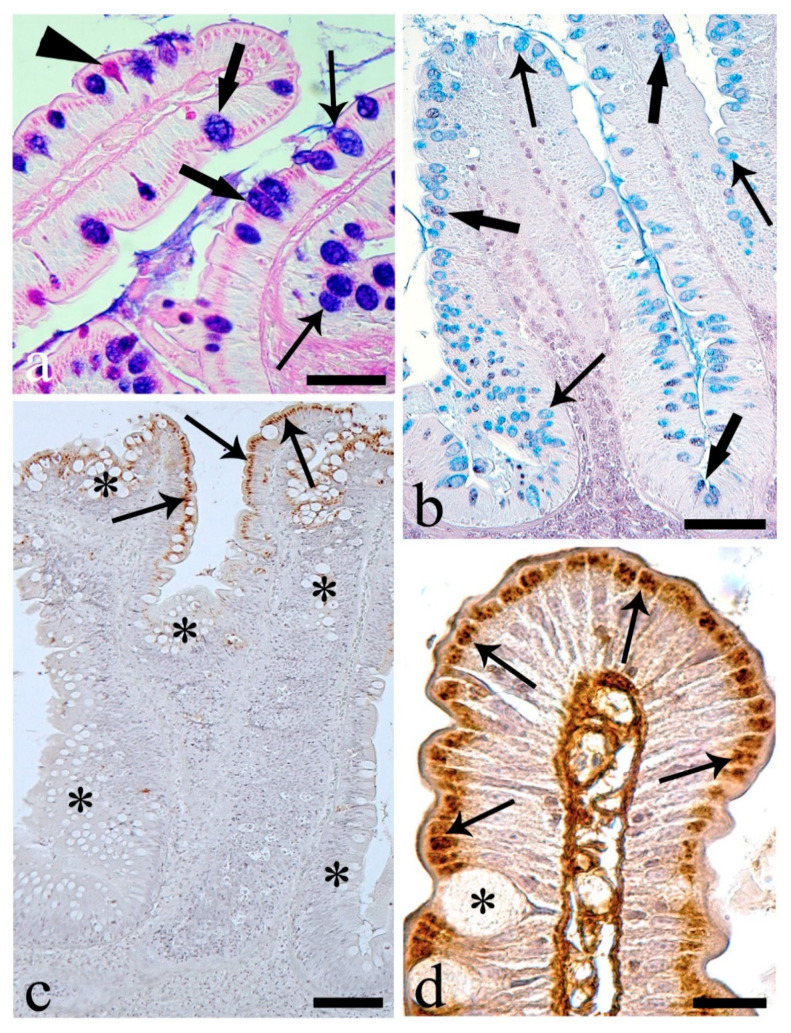
Proximal intestine. (**a**) Mucous cells containing mainly AB-positive mucins (thin arrows), AB/PAS-reactive mucins (thick arrows), and only few PAS-positive mucins (arrowhead). Scale bar: 50 μm. (**b**) Intestinal folds with predominant AB-positive mucous cells (thin arrows), and few HID-reactive mucous cells (thick arrows). Scale bar: 100 μm. (**c**) Positive reaction to lectin DBA in supra-nuclear cytoplasm of enterocytes at the tip of the folds (thin arrows). Unreactive mucous cells (asterisks). Scale bar: 100 μm. (**d**) Supra-nuclear cytoplasm of enterocytes (thin arrows) reactive to the lectin WGA and mucous cells unreactive (asterisks). Scale bar: 20 μm.

In the proximal intestine, AB-positive mucous cells were most abundant, with PAS-positive being less frequent (Figure 4a, Table 4). Many mucous cells contained carboxylated acid mucins (AB-positive); few were HID-positive, with sulfated acidic mucins (Figure 4b, Table 5). All mucous cells were nonreactive to the lectins, although, at the apex of the intestinal folds, strong reactivity to DBA (Figure 4c) and WGA (Figure 4d) lectins was evident at the apices of the enterocytes. 

**Figure 5:**
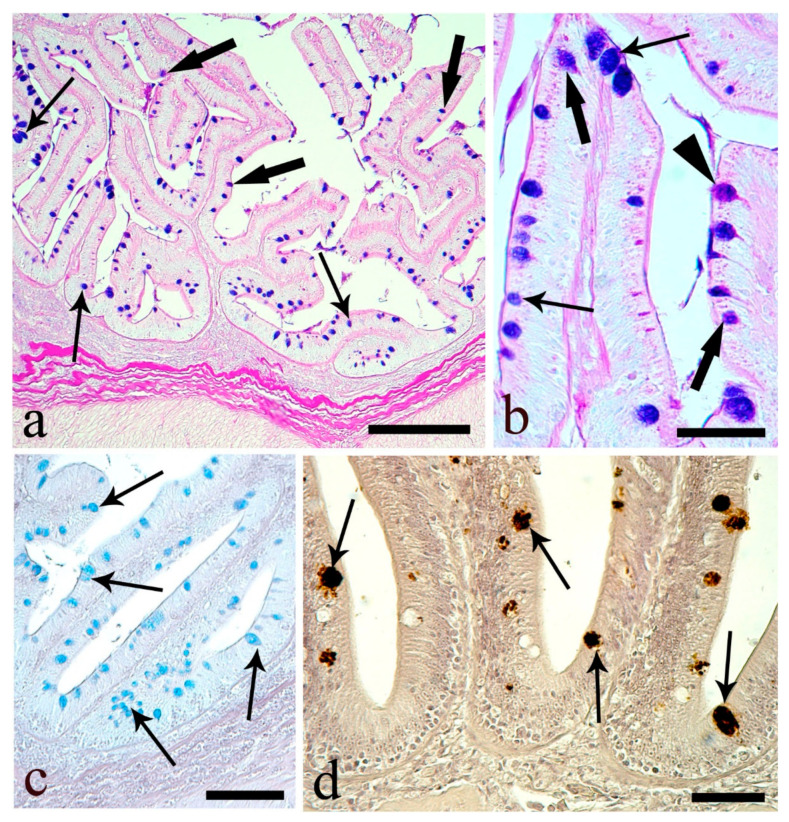
Medial intestine. (**a**) *Tunica mucosa* raised in anastomotic folds rich in mucous cells positive to AB (thin arrows) and AB/PAS (thick arrows). Scale bar: 200 μm. (**b**) Three types of mucous cells: AB (thin arrows)-, PAS- (arrowhead)-, and AB/PAS (thick arrows)-positive cells. Scale bar: 50 μm. (**c**) Mucous cells containing mainly carboxylated mucins (thin arrows). Scale bar: 100 μm. (**d**) Mucous cells positive to lectin WGA (thin arrows). Scale bar: 50 μm.

The distribution of mucous cells in the medial intestine was almost identical to that of the proximal region, with a lower number of AB-positive cells and an increase of PAS-positive mucous cells (Figure 5a,b, Table 4). The medial intestine possessed the lowest mean number of mucous cells (Figure 5a, Table 4 and Table 5). The number of HID-positive mucous cells was low, and it was likely that all acid mucins were of the carboxylated type (Figure 5c, Table 5). In the medial intestine, PNA was the only lectin type detected (Figure 5d). The number of mucous cells per 100,000 µm^2^ positive to PNA was 33.0 ± 1.2, 44.2% of the total mucous cells.

**Figure 6:**
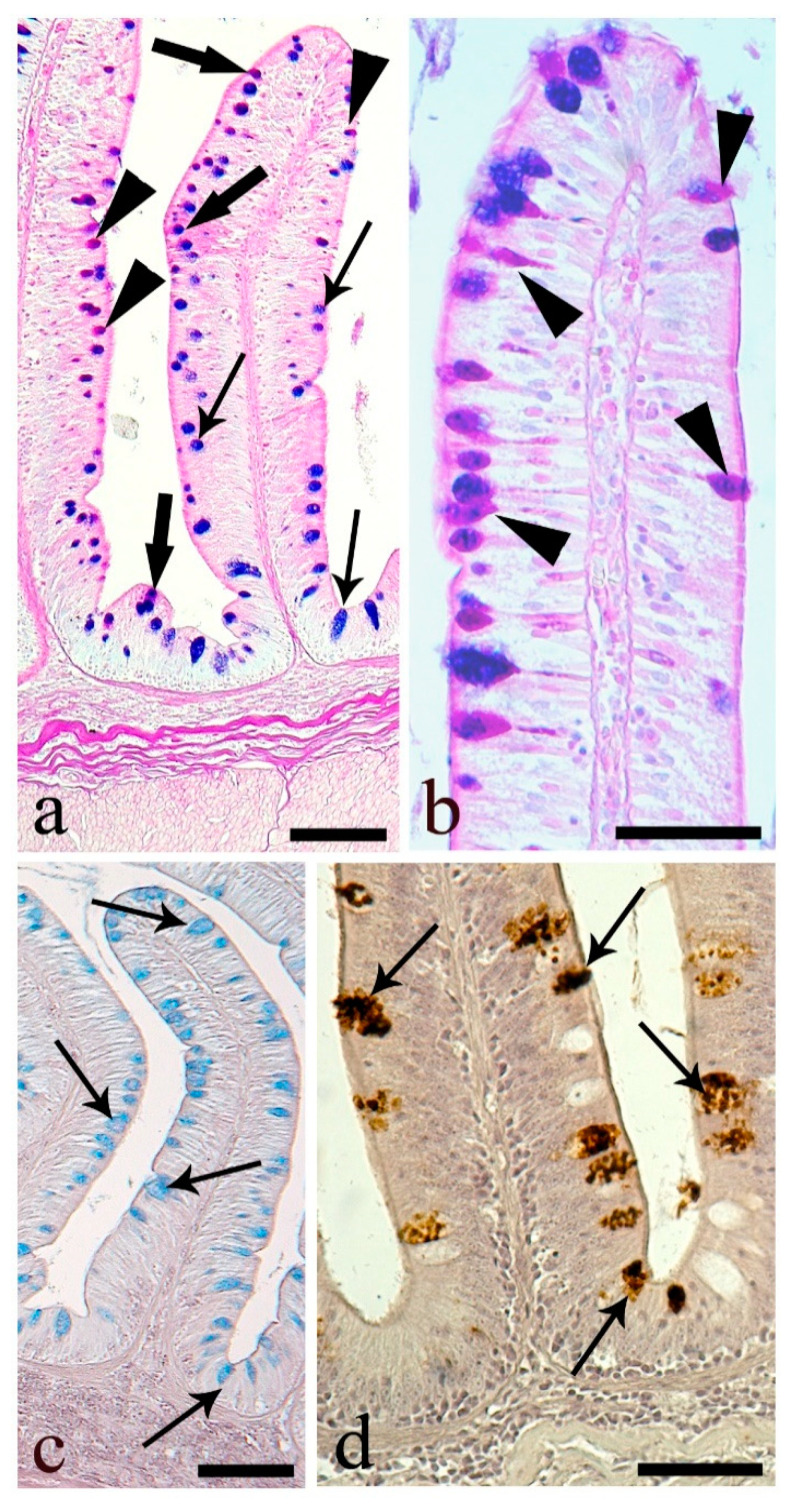
Distal intestine. (**a**) *Tunica mucosa* showing elongated not anastomotic folds containing numerous mucous cells: AB- (thin arrows), AB/PAS- (thick arrows), and PAS-positive (arrowhead). Scale bar: 100 μm. (**b**) PAS-positive cells mainly at the top of the intestinal folds (arrowheads). Scale bar: 50 μm. (**c**) AB-positive mucous cells (thin arrows) and no sulfated mucous cells. Scale bar: 100 μm. (**d**) Numerous cells positive to lectin PNA (thin arrows). Scale bar: 50 μm.

The PAS-positive mucous cells in distal intestine were twice that in medial, 24.8 vs. 12.4 per 100,000 µm^2^, and the AB-positive cells lower at 52.0% vs. 41.6% of the total mucous cells in medial vs. distal intestine (Figure 6a,b, Table 4). In the distal intestine, PAS-positive mucous cells were numerous at the top of the folds (Figure 6b), with no HID-positive mucous cells detected (Figure 6c, Table 5). Approximately half of the mucous cells showed reactivity only to the lectin PNA (Figure 6d). The number of mucous cells per 100,000 µm^2^ reactive to PNA was 54.0 ± 2.4, 51.0% of the total mucous cells in the distal intestine.

**Figure 7:**
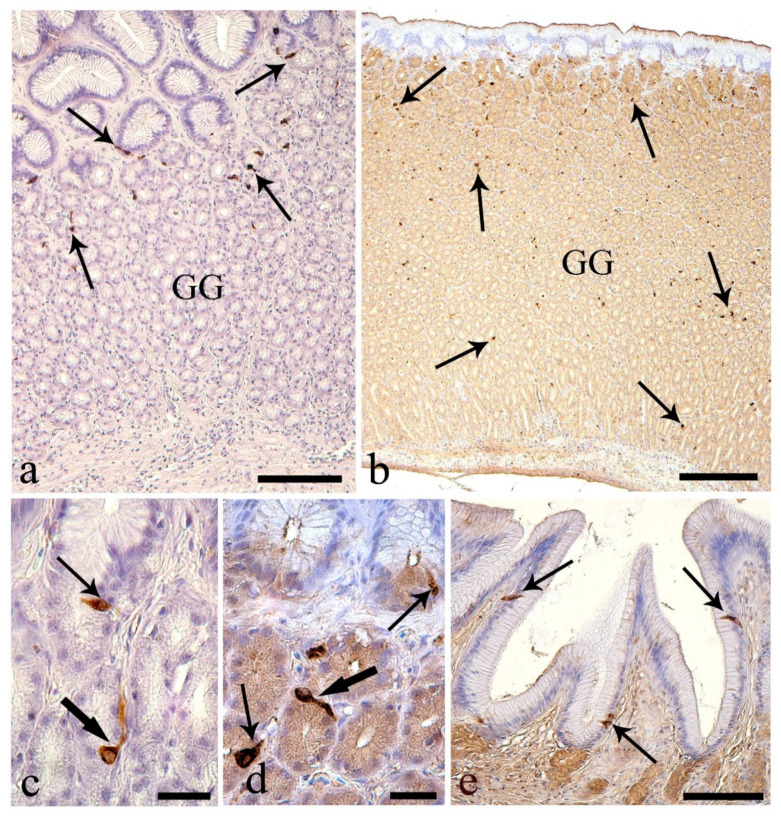
The stomach. (**a**) Several endocrine cells in the upper stomach gland layer were immunoreactive to anti-tyrosine hydroxylase (thin arrows); GG: gastric glands. Scale bar: 100 μm. (**b**) Numerous endocrine cells in gastric glands immunoreactive to antibody anti-somatostatin-14 (thin arrows). Scale bar: 200 μm. (**c**) Closed type endocrine cells with cytoplasmatic process (thick arrow), and an open type endocrine cell (thin arrow) both reactive to anti-tyrosine hydroxylase. Scale bar: 20 μm. (**d**) Anti-somatostatin-14 positive open type (thin arrows) and closed type endocrine cells with cytoplasmic process (thick arrow). Scale bar: 20 μm. (**e**) Many endocrine cells immunoreactive to anti-substance P (thin arrows). Scale bar: 100 μm.

**Figure 8:**
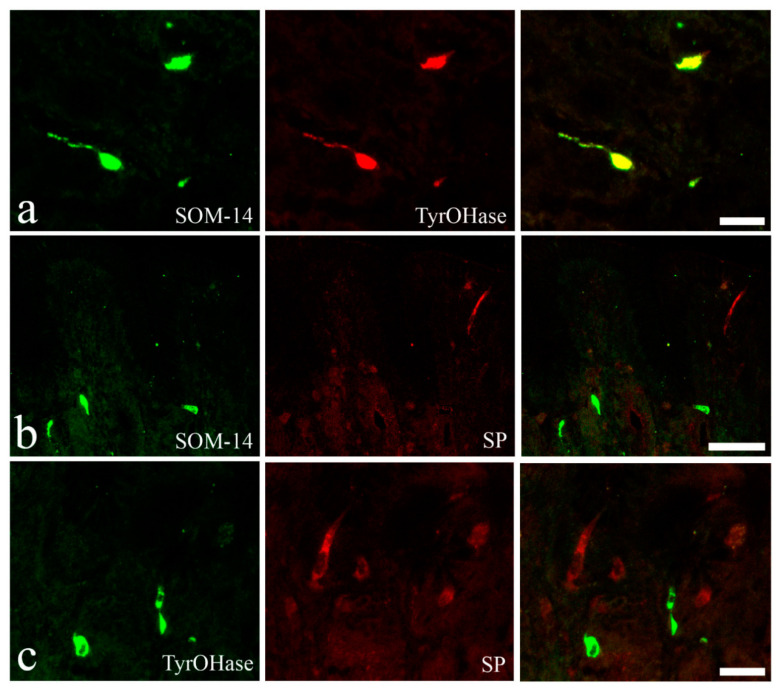
Co-localization tests with confocal laser-scanning microscopy. Each row represents a double immunofluorescence reaction, with the same microscopic field showing endocrine cells immunoreactive to the first primary antibody tested, to the second primary antibody used, and superimposition of the two images. (**a**) Gastric glands: the same endocrine cells were immunoreactive to anti-somatostatin-14 (SOM-14) and anti-tyrosin hydroxylase (Tyr-OHase). Scale bar: 20 μm. (**b**) The stomach: endocrine cells immunoreactive to anti-somatostatin-14 (SOM-14) and those positive to anti-substance P (SP) differed. Scale bar: 50 μm. (**c**) Gastric mucosa: endocrine cells immunoreactive to anti-tyrosine hydroxylase (TyrOHase) and those reactive to anti-SP (SP) differed. Scale bar: 20 μm.

**Figure 9:**
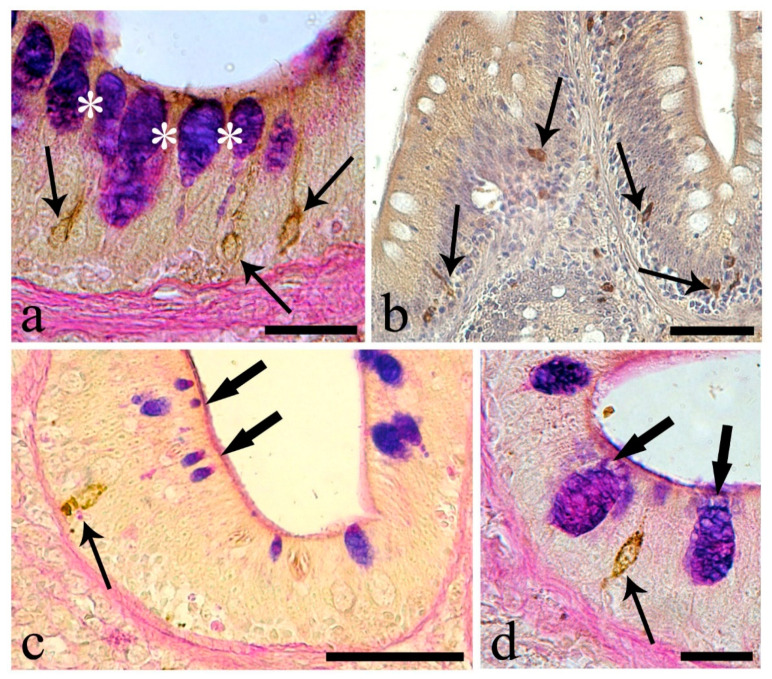
Intestine. (**a**) Three endocrine cells of proximal intestine reactive to anti-substance P (thin arrows) close to a group of mucous cells mainly AB/PAS-positive (asterisks). Scale bar: 20 μm. (**b**) Anti-Leu-enkephalin immunoreactive endocrine cells (thin arrows) in medial intestine. Scale bar: 50 μm. (**c**) Base of the medial intestinal epithelium; an endocrine cell positive to anti-Leu-enkephalin (thin arrow) in correspondence of different mucous cells that were AB/PAS-positive (thick arrows). Scale bar: 50 μm. (**d**) Endocrine cell immunoreactive to anti-Leu-enkephalin (thin arrows) close to AB/PAS-positive (thick arrows) mucous cells in distal intestine. Scale bar: 20 μm.

In the stomach, a high number of endocrine cells immunoreactive to the antibody anti-tyrosine hydroxylase (TH) were present in the upper half of the gastric mucosa (Figure 7a). Endocrine cells immunoreactive to anti-somatostatin-14 (SOM) were observed throughout the glandular layer of the *E. lucius* stomach (Figure 7b). The TH-immunoreactive and SOM-immunoreactive endocrine cells were scattered among the cells of the glandular acini, with some exhibiting a thin cytoplasmic extension along the basal membrane (Figure 7c,d). The mean numbers of endocrine cells per 100,000 µm^2^ positive to anti-TH and -SOM were 29.7 ± 1.1 and 22.6 ± 1.0, respectively. In the stomach, a low number of endocrine cells (4.4 ± 0.4 per 100,000 µm^2^) scattered among cells of the surface epithelium were immunoreactive to anti-substance P (Figure 7e). The endocrine cells in the upper half of the gastric mucosa co-localized anti-SOM and -TH (Figure 8a). In contrast, endocrine cells positive for anti-substance P were not co-localized with anti-SOM or anti-TH (Figure 8b,c). A lower number of epithelial endocrine cells immunoreactive to anti-substance P was observed in the proximal intestine (2.8 ± 0.2 per 100,000 µm^2^) compared with in the stomach. Substance P-immunoreactive endocrine cells of the proximal intestine were often near a single mucous cell or a group of mucous cells (Figure 9a).

The three intestinal areas showed endocrine epithelial cells immunoreactive to anti-Leu-enkephalin (Figure 9b–d) with a progressive increase in mean numbers: 1.2 ± 0.1 in the proximal, 2.0 ± 0.2 in the medial, and 3.7 ± 0.2 in the distal intestine per 100,000 µm^2^. Leu-enkephalin-IR endocrine cells were observed near one or more mucous cells in the epithelium of the intestine (Figure 9c,d).

## 4. Discussion

In this study, pikes were collected in the spring of 2019 and the mucosal features reflected the morphological aspects of springtime. Indeed, García-Meilán et al. [21] showed seasonal differences with higher digestive enzyme synthesis and food intake in spring, due to the longer photoperiod in comparison to autumn. In winter, wild fish can stand fasting for a relatively long time lapse, with changes of gut morphology and a reduction of 20–75% of digestive enzymatic activity [22,23].

The alimentary tract of *E. lucius* consists of a short esophagus that is contiguous with a wider stomach and the intestine [2]. The histometric measurements on the mucosal features of the different gut regions showed the higher epithelial height in the esophagus and the higher mucosal folds in the intestine. The measures enabled future comparisons with studies on experimental conditions and/or on evaluation of possible pathologies of the alimentary canal of pike. The epithelial mucous cells of the gut secrete mucins, influencing feeding efficiency [24] and playing an important role in digestive physiology [25,26,27]. Most studies of the distribution of mucous cell types in the gastrointestinal tract of fish have reported only presence/absence data [6,7,27,28,29,30]. Mucous cell counts allow a morphology/function comparison of parts of the digestive tract [8].

### 4.1. Esophagus

The esophageal mucosa of *E. lucius* shows longitudinal folds to facilitate the transit of large food items [1]. In the epithelium, the abundance of mucous cells (≈27% of the mucous cells in the alimentary canal), and their variable mucin composition can be reflective of the lack of salivary glands [31]. In the *E. lucius* esophagus, the predominance of mucous cells containing neutral mucins is related to pre-gastric digestion [32]. The dual function of the esophagus in food transfer and pre-digestion has been reported in other carnivorous fish species [5,10,11,27,28,29,33].

The quantity of mucous cells containing acidic or mixed mucins in the *E. lucius* esophagus was similar. Of the acid mucins, 57.6% were carboxylated and 42.4% sulfated. In the oral cavity and esophagus of the Mrigal carp (*Cirrhinus mrigala*), the carboxylated mucins prevent micro-organism adherence to the epithelial surface [34]. In addition, carboxylic acid residue in acid mucins is considered a scavenger of reactive oxygen species, which can be generated by the ingestion of pollutants from the environment [26]. The increased mucus viscosity due to sulfated mucins helps to trap small particles and aggregate them into the food bolus [10,35,36].

Over 50% of mucous cells in the esophagus of *E. lucius* were positive for WGA lectin, and 17.9% were positive for DBA. In shi drum (*Umbrina cirrosa*) larvae, WGA has been reported in esophageal mucous cells, indicating the presence of sialic acid and N-acetyl-glucosamine residues [24]. The DBA lectin binds to N-acetyl-galactosamine residues and has also been reported in esophageal mucous cells of *U. cirrosa* larvae [24] and gilthead bream (*Sparus aurata*) larvae and adults [33]. In neutral mucins, the presence of N-acetyl-galactosamine residues is related to ion and fluid transport across the plasmalemma [33,37] and is involved in enzymatic digestion [24,38]. The reactivity of the epithelial brush border to ConA lectin revealed the presence of mannose or glucose residues, indicating carbohydrate absorption [39]. A weak reaction to ConA was detected in the esophagus of the stripped weakfish (*Cynoscion guatucupa*) [5] and throughout the alimentary canal of the common dentex (*Dentex dentex*), indicating carbohydrate absorption in the entire gut [25].

### 4.2. Stomach

In *E. lucius*, the junction of the esophagus and stomach is not macroscopically visible but is recognizable microscopically by abrupt change in the epithelium and the presence of gastric glands [1]. The stomach mucosa is characterized by columnar epithelium interspersed with gastric pits that extend into the gastric glands. *E. lucius* gastric glands are mainly alveolar, generally associated with lower acid secretion when compared with the tubular gastric glands of the distal stomach [1].

The study of Bucke [2] reports the presence of columnar cells with neutral mucins in the stomach epithelium of *E. lucius*, with a few AB-stained cells at the base of the pits, a state we observed only in the area of transition from the esophagus to stomach mucosa. The columnar cells of the stomach contain primarily mixed mucins, with the acid component exclusively carboxylated [3]. The presence of columnar cells with both neutral and acid carboxylated mucins is reported in the stomach of the European eel (*Anguilla anguilla*) [10] and adult *S. aurata* [33]. In general, acid mucins prevent damage to stomach epithelium by acting as a lubricant [40,41]. In the stomach, neutral mucins form a physical viscoelastic gel to protect the mucosal layer against injury by enzymes and hydrochloric acid produced in gastric glands [33,38,40,42]. In *E. lucius*, the neutral mucins of the columnar cells are characterized mainly by galactosyl(b1-3)-N-acetylgalactosamine residues, a finding also reported in eel [10]. The neutral mucins secreted in the stomach are related to carbohydrate and short-chain fatty acid absorption through the plasma membrane [3,24,30,43,44]. The gastric glands showed no reaction to the AB/PAS and HID/AB sequence, similar to results of other investigated species [30,41].

Several processes in the gut are locally regulated by enteroendocrine cells (EC) of the diffuse endocrine system scattered among enterocytes [19,45,46]. A stimulus originating from within the gut lumen or from tissue cells causes hormone release from the basolateral membrane of ECs, often acting as a paracrine signal [16,45]. In the stomach of vertebrates, the main regulatory molecules of gastric acid secretion are gastrin, somatostatin, and the catecholamines L-3,4-dihydroxyphenylalanine (L-DOPA) and dopamine (DA) [45,47]. In the stomach of *E. lucius*, several ECs immunoreactive to anti-SOM and -TH (the enzyme involved in the conversion from tyrosine to L-DOPA) were observed in the gastric glands. We subjected stomach tissue of *E. lucius* to two anti-gastrin antibodies with negative results (data not shown). In fish with a piscivorous diet such as the pike, the presence of gastrin-immunoreactive ECs was controversial, because it was reported in the stomach of the Dorado (*Salminus brasiliensis*) [48] but not in the stomach of the largermouth bass (*Micropterus salmoides*) and the northern snakehead (*Channa argus*) [49].

Somatostatin acts directly on parietal and gastrin ECs to inhibit gastric acid secretion [45,47]. As in mammals, SOM ECs of *E. lucius* exhibit cytoplasmic processes reaching the neighboring cells along the basal membrane [50]. Similarly, SOM ECs have been documented in the stomach of the milkfish (*Chanos chanos*) [51] and the predatory longnose gar (*Lepisosteus osseus*) [52].

In the gastrointestinal tract of mammals, the main source of secreted and circulating L-DOPA and DA is the stomach [53,54]; therefore, TH is expressed in ECs of the gastric mucosa [53,55]. We observed several ECs related to the biosynthesis of L-DOPA that are immunoreactive to anti-TH. Experimental studies of rats demonstrated that gastric catecholamines decrease and increase during fasting and feeding, respectively [56]. In goldfish (*Carassius auratus*), experimental hormonally induced fasting/feeding showed a direct influence on the catecholamine synthesis pathway [57]. In the stomach of *E. lucius*, SOM and TH were co-localized in the ECs of the glandular layer, indicating a dual regulatory function of these ECs. Conversely, anti-substance P did not co-express with SOM or TH antibodies in the ECs of *E. lucius* stomach, indicating the existence of different EC types. The activation of genetic switches can lead to the expression of a given neuroendocrine phenotype in the same cell [58]. Thus, it may be that the ECs of *E. lucius* stomach are poly-functional, and different stimuli might induce the release of a specific marker.

Enteroendocrine cells immunoreactive to anti-substance P are found in the epithelium of the *E. lucius* stomach. This neuropeptide was reported in ECs in the stomach of rainbow trout (*Oncorhynchus mykiss*) [59] and *S. aurata* [60]. In the gut of European seabass (*Dicentrarchus labrax*) fry, ECs immunoreactive to anti-substance P appeared at four days post-hatching, evidence for an important role in the development of the gut epithelium [61].

### 4.3. Intestine

In *E. lucius*, histology of the intestinal mucosa revealed aspects related to its carnivorous diet. The proximal intestine showed a higher number of mucous cells compared to the medial and distal parts. In most fish, the abundance of mucous cells increases from proximal to distal in the intestine [8,10,27]. Acid mucins are predominant in the proximal intestine of *E. lucius*, the only region where a low number of mucous cells containing acidic sulfated mucins was identified. In fish, sulfated mucins protect the intestinal mucosa from the gastric acid chime [62] and regulate the transport of proteins and their fragments, as well as ions and fluids, across the plasma membrane [33,42,62]. We detected mucous cells containing sulfated mucins in the proximal intestine, whereas carboxylated mucins were present in proximal, medial, and distal regions. In most fish, the secretion of sulfated mucins increases from proximal to distal in intestine [9]. The abundant carboxylated mucins in the intestine of the grass carp (*Ctenopharingodon idella*) induces rapid gut transit of the digesta [9], increasing efficiency of elimination of food residue [6,63].

The presence of neutral mucins is associated with digestive and absorptive processes [6]. Neutral mucins in the intestine of *E. lucius* showed an increase from 3.8%, 12.9%, to 22.3% in the proximal, medial, and distal region, respectively. Digestion and absorption are likely increased from proximal to distal intestine of *E. lucius*, maximizing the feeding efficiency in spite of a short gut with a relatively rapid transit.

In the proximal intestine of *E. lucius*, DBA and WGA mark the supranuclear cytoplasm of the enterocytes. Reactivity to DBA was reported in enterocytes of *O. mykiss* [11] and intestine of *A. anguilla* [10]. These sugar residues play a role in the enzymatic digestion and transformation of food in chime [24,33,38,42]. In mammalian enterocytes, N-acetyl-galactosamine is also involved in the regulation of fluid and ion movement [24,33,37]. In the present study, the percent of mucous cells reactive to PNA was 44.2 in the medial and 51.0 in the distal intestine. A similar finding was reported with regard to PNA-positive mucous cells in the intestine of poly-hybrids of *Tilapia* spp. [64].

A decreasing trend in Leu-enkephalin-immunoreactive ECs was observed in the intestine of *C*. *chanos* [51] and the South American catfish (*Rhamdia quelen*) [65]. We observed that ECs immunoreactive to anti-Leu-enkephalin were few and increased from proximal to distal in intestine of *E. lucius*. In teleosts, opioids are reported in ECs, where they regulate intestinal motility and are involved in the modulation of immune system cells [46,66]. Mucus discharge from intestinal mucous cells is induced by EC secretion of opioids following luminal stimuli [19,67]. In the intestine of *E. lucius*, the increase of Leu-enkephalin-immunoreactive ECs was possibly related to the reduction in number of mucous cells from the proximal to the distal intestine.

### 4.4. Conclusions

In *E. lucius*, as in other carnivorous fish, the digestion of food begins in the esophagus where mucous cells containing neutral mucins are numerous. This is not the case in herbivorous or omnivorous fish [6,34]. The distribution of mucous cell types in the fish digestive tract is reflective of their feeding behavior [8]. In the stomach, there is a high abundance of mixed mucins, with the acid component contributing to the lubrication of mucosae, aiding transit of the alimentary bolus [40]. Carboxylated acid mucins predominate in the intestine, where they are associated with the rapid passage of digesta through the intestine [6,9,63]. Neutral mucins increase in the intestine aborally, in tandem with an expansion of nutrient absorption [10,28,29,33,68]. The distribution of ECs of the diffuse endocrine system shows the presence of SOM- and catecholamine-secreting ECs and the lack of gastrin-secreting ECs [49,51,57]. These factors enhance the feeding efficiency of *E. lucius*, a fish with a short gut and rapid transit of digesta.

## Figures and Tables

**Table 1:** Biotinylated lectins used in this study, their sources, and carbohydrate binding affinity.

Acronym	Vector Laboratories Code	Lectin	Species Source: Latin Name (Common Name)	Major Carbohydrate Specificity
ConA	B-1005	Concanavalin A	*Canavalia ensiformis* (Jack bean)	α-Mannose, α-Glucose
DBA	B-1035	Dolichos biflorus agglutinin	*Dolichos biflorus* (horse gram)	α-GalNAc
PNA	B-1075	Peanut agglutinin	*Arachis hypogaea* (peanut)	Gal β 1-3GalNAc
DSL	B-1185	Datura stramonius lectin	*Datura stramonium* (thorn apple)	(GlcNAc)n, Gal β 1-4GlcNAc
WGA	B-1025	Wheat germ agglutinin	*Triticum vulgare* (wheat germ)	(GlcNAc)n, Sia
UEA I	B-1065	Ulex europaeus agglutinin I	*Ulex europaeus* (gorse seed)	α-Fucose

GalNAc: N-acetylgalactosamine; Gal β 1-3GalNAc: galactosyl β 1-3 N-acetylgalactosamine; GlcNAc: N-acetylglucosamine; Gal β 1-4GlcNAc: galactosyl β 1-4 N-acetylglucosamine; Sia: sialic acid; Gal: galactose.

**Table 2:** Antibodies used on the alimentary canal sections of *Esox lucius*.

Antibody Anti-	Clonality	Host	Source, Code	Dilution and Incubation at Room Temperature
Somatostatin-14	Polyclonal	Rabbit	Genosys Biotechnologies Inc., Cambridge, UK, CA-08-325	1:200; 24 h
	Monoclonal	Mouse	Santa Cruz Biotechnology Inc., Santa Cruz, CA, USA, sc-74556	1:50; 24 h
Substance P	Polyclonal	Rabbit	Peninsula Labs. Int., Belmont, CA, USA, T-4170	1:200; 24 h
	Monoclonal	Mouse	Santa Cruz Biotechnology Inc., Santa Cruz, CA, USA, sc-14184	1:50; 24 h
Leu-enkephalin	Polyclonal	Rabbit	Peninsula Labs. Int., Belmont, CA, USA, IHC 8601	1:500; 24 h
	Monoclonal	Mouse	Santa Cruz Biotechnology Inc., Santa Cruz, CA, USA, sc-47705	1:200; 24 h
Tyrosine hydroxylase	Polyclonal	Rabbit	Millipore, Burlington, MA, USA, AB152	1:250; 24 h
	Monoclonal	Mouse	Santa Cruz Biotechnology Inc., Santa Cruz, CA, USA, sc-25269	1:50; 24 h
Anti-biotinylated secondary antibodies				
Anti-rabbit IgG		Goat	Vector Labs, Burlingame, CA, USA, BA-1000	1:1000; 2 h
Anti-mouse IgG		Goat	Vector Labs, Burlingame, CA, USA, BA-9200	1:1000; 2 h

**Table 3:** Morphometric parameters (µm) ± standard error (SE) of the main mucosal features in the alimentary canal of *Esox lucius*. Each data point was the result of five measures, 40× objective magnification, from three slides in the 10 specimens (total 150 measures).

Morphometric Parameters	Esophagus	Stomach	Intestine		
			Proximal	Medial	Distal
MFsH	198.5 ± 4.2	163.2 ± 3.8	480.2 ± 15.0	536.1 ± 15.0	575.7 ± 11.6
MFsW	167.3 ± 4.1	83.1 ± 2.7	123.3 ± 1.8	75.0 ± 1.3	123.1 ± 2.9
EpH	66.3 ± 1.5	39.3 ± 1.1	54.7 ± 1.5	33.7 ± 0.8	44.1 ± 1.00

MFsH = mucosal folds height, MFsW = mucosal folds width, EpH = epithelial height.

**Table 4:** Mean ± SE of mucous cells per 100,000 µm^2^ of epithelium in the alimentary canal of *Esox lucius* containing acid mucins. Mucous cells were counted in five fields, 40× objective magnification from three slides in each of 10 specimens (total of 150 microscopic fields).

Mucous Cells	Esophagus	Stomach	Intestine		
			Proximal	Medial	Distal
AB	97.6 ± 2.8	-	97.7 ± 2.5	49.6 ± 1.0	46.3 ± 1.7
PAS	199.2 ± 5.3	230.0 ± 7.1	6.3 ± 0.4	12.4 ± 0.4	24.8 ± 1.2
AB/PAS	99.4 ± 2.8	504.2 ± 10.0	61.9 ± 2.3	33.6 ± 0.7	40.1 ± 1.1
Total	377.6 ± 6.8	731.2 ± 13.6	167.7 ± 2.7	92.9 ± 1.1	109.3 ± 1.7

AB = alcian blue, PAS = periodic acid Schiff, AB/PAS = mixed.

**Table 5:** Mean ± SE of mucous cells per 100,000 µm^2^ of epithelium in the alimentary canal of *Esox lucius* containing carboxylated (AB-positive) and sulfated (HID-positive) mucins. Mucous cells were counted in five fields, 40× objective magnification from three slides in each of 10 specimens (total of 150 microscopic fields).

Mucous Cells	Esophagus	Stomach	Intestine		
			Proximal	Medial	Distal
AB	233.8 ± 6.3	710.7 ± 10.1	140.5 ± 3.7	78.4 ± 2.5	95.7 ± 1.4
HID	172.3 ± 4.9	-	29.8 ± 1.4	0.9 ± 0.1	-
Total	405.9 ± 8.2	710.7 ± 10.1	170.3 ± 4.1	79.3 ± 2.5	95.7 ± 1.4

AB = alcian blue, HID = high iron diamine.
